# A smart tool for non expert clinicians for the dissemination of the MDS criteria for progressive supranuclear palsy

**DOI:** 10.1007/s10072-025-07996-0

**Published:** 2025-01-11

**Authors:** Marina Picillo, Filomena Abate, Vincenzo Canoro, Maria Francesca Tepedino, Paolo Barone, Roberto Erro

**Affiliations:** 1 Center for Neurodegenerative Diseases (CEMAND), Department of Medicine, Surgery and Dentistry, “Scuola Medica Salernitana”, University of Salerno, Salerno, Italy; 2 Department of Neurology, “Umberto I” Hospital, Nocera Inferiore, Salerno, Italy; 3 UOC Clinical Neurologica, AOU San Giovanni di Dio e Ruggi d’Aragona, Salerno, Italy; 4 IRCCS SynlabSDN, Naples, Italy

**Keywords:** Progressive supranuclear palsy, MDS criteria, Diagnosis, Phenotype

## Abstract

Due to the variety of clinical phenotypes and the massive clinical overlap with other neurodegenerative diseases, the diagnosis of Progressive Supranuclear Palsy (PSP) remains a major challenge. Notwithstanding, early and reliable clinical diagnosis of PSP is highly warranted for estimation of prognosis, appropriate allocation to therapeutic trials and development of new diagnostic tools. As reliable biomarkers are lacking, PSP diagnosis relies on the application of the clinical criteria promoted by the International Parkinson and Movement Disorder Society (MDS). Despite providing a framework including all the main PSP cornerstones (ocular dysfunction and postural instability, akinesia and cognitive dysfunction), the application of the MDS PSP criteria is complex and not straightforward to apply in a clinical setting. Herein we propose a practical tool, including a video-guided slide-set and a smartsheet, to disseminate the MDS PSP clinical criteria among healthy practitioners and increase confidence in non expert clinicians towards suspicion and diagnosis of PSP. The video-guided slide set may serve as a teaching resource for both general neurologists and practitioners, while the smartsheet may represent a valid support in attributing the degree of diagnostic certainty and phenotype based on the identified clinical features. Application of our tool may improve early recognition of patients in primary and secondary care and determine a prompt referral to third level movement disorder centers for consideration in clinical trials testing disease-modifying treatments.

**Supplementary Information:**

The online version contains supplementary material available at 10.1007/s10072-025-07996-0.

## Introduction

Progressive supranuclear palsy (PSP) is a rare, rapidly progressive, neurodegenerative 4R-tauopathy, characterized by ocular motor dysfunction, postural instability, akinesia and cognitive dysfunction [1]. The diverse combination of the core clinical features is determinant for the attribution of the clinical phenotype of the disease [1]. While PSP Richardson’s syndrome (PSP-RS) is the most common clinical phenotype, other distinct variants of the disease (vPSP) have been described, each featured by a specific predominant symptom [1]. As for other neurodegenerative diseases, definite diagnosis of PSP relies on neuropathological criteria [2]. Indeed, due to its dynamic nature, various clinical phenotypes, and similarities to other neurologic disorders, the clinical diagnosis of PSP remains a major challenge. Notwithstanding, early and reliable clinical diagnosis of PSP is paramount for increasing the knowledge of the disease, estimation of prognosis, appropriate allocation to therapeutic trials and development of new diagnostic tools.

Recently, the International Parkinson and Movement Disorder Society (MDS)-endorsed PSP Study Group provided an evidence- and consensus-based revision of the original National Institute of Neurological Disorders and Stroke and Society criteria for PSP (NINDS-SPSP) [1, 3]. Indeed, in spite of being straightforward and relatively simple to apply in the clinical setting, the NINDS-SPSP criteria were unbalanced towards PSP-RS as strongly based on the presence of vertical supranuclear gaze palsy and postural instability and lacked in sensitivity especially for vPSP.

Based on the identification of four core clinical domains, the MDS PSP criteria provide different degree of diagnostic certainty and allow the attribution of the clinical phenotype [1]. However, given the frequent overlap between different phenotypes, the Multiple Allocations eXstinction (MAX) rules were conceived to establish a precise hierarchy among the phenotypes and to guide the clinician in reaching the correct diagnosis [4]. Despite being more accurate, the MDS PSP criteria are complex to apply especially for non expert in movement disorders and in real-world clinical settings.

Aim of the present review is to provide a clear and straightforward framework for non expert clinicians for the application of the MDS PSP criteria. Being useful also for teaching purposes, such tool may be of support for the dissemination strategy of the MDS PSP criteria and pivotal to increase awareness of this rare disease in the broader medical community.

Our framework include:

1) a video-guided slide set showing all degrees of dysfunction for each core domain with demonstrative videos for the application of the MAX rules;

2) a user-friendly smartsheet to support clinicians in the correct attribution of the degree of diagnostic certainty and phenotype.

## Methods

All pictures and videos were extracted from the archive of the PSP outpatient clinic of the Center for Neurodegenerative Diseases (CEMAND) at the University of Salerno, Italy [5, 6]. All patients had previously provided written informed consent to taping and publication of their videos. Each recording was evaluated by two movement disorder experts (MP, RE) for quality control and selection of the clinical signs to be included in the slide set as teaching content. The criteria for the choosing process were: (1) presence of a close-up on the patient and on the clinical sign of interest; (2) good light conditions; (3) representativeness of the clinical sign, so that each video corresponds either to one level of certainty from a functional domain of the MDS criteria, or to a phenotype of the disease. For each slide a comment is provided by an English native speaker.

The showed videos have been cut and adapted to the slides with QuickTime Player 7.6.6 for Mac OS X v10.6.3, and the final slide set has been created with Microsoft office PowerPoint for Mac 2011 version 14.6.6 and iMovie 10.1.16 for Mac OS X v10.15.7.

MDS diagnostic criteria and MAX rules were used for the smartsheet creation. The final file has been created with Microsoft Office Excel for Mac 2011 version 14.6.6.

### Video-guided slide set

Four core functional domains represent characteristic clinical manifestations of PSP [ocular motor dysfunction (O), postural instability (P), akinesia (A), and cognitive dysfunction (C)]. For each domain, three characteristic core clinical features are proposed, stratified by presumed levels of certainty (1- the highest, 2- the intermediate, and 3- the lowest) contributing to the diagnosis of PSP (Table 1). The following sections summarize the operationalized definitions of the core clinical features provided by the MDS PSP criteria and are intended to accompany Video 1 [1].

**Table 1:** Definitions of clinical features for all levels of diagnostic certainty and core domains from the PSP diagnostic criteria

Levels of certainty	Ocular motor dysfunction (O)	Postural instability (*P*)	Akinesia (A)	Cognitive dysfunction (C)
1 (Highest)	**VSGP** Limited range of voluntary gaze on the vertical plane with preserved VOR	**Repeated unprovoked falls** ^*^ Spontaneous loss of balance or more than one unprovoked fall	**Progressive gait freezing** ^*^ Sudden and transient motor blocks or start hesitation	**nfaPPA** or **Apraxia of speech** Effortful, halting speech with frequent sound errors
2	**Slow velocity of vertical saccades** Decreased velocity and amplitude of vertical saccadic movements	**Tendency to fall on the pull-test** ^*^ If not caught by examiner	**Parkinsonism**,** akinetic-rigid**,** axial-predominant**,** LD-resistant** Bradykinesia and rigidity axial-predominant	**Frontal presentation** At least three of the following: 1) Apathy 2) Bradyphrenia 3) Dysexecutive syndrome 4) Reduced phonemic verbal fluency 5) Impulsivity, disinhibition, perseveration
3 (Lowest)	**MSWJ** Rapid, involuntary saccadic intrusions on the horizontal plane or **Eyelid opening apraxia** Inability to initiate eyelid opening after lid closure	**More than 2 steps on the pull-test** ^*^ but unaided recovery	**Parkinsonism with tremor and/or asymmetric and/or LD-responsive**	**CBS** At least one sign for each category **Cortical signs** 1) Orobuccal or limb apraxia 2) Cortical sensory deficit 3) Alien limb phenomena **Movement disorder signs** 1) Rigidity 2) Akinesia 3) Myoclonus

Modified from Hoglinger GU, Respondek G, Stamelou M et al., for the Movement Disorder Society–endorsed PSP Study Group (2017) Clinical Diagnosis of Progressive Supranuclear Palsy: The Movement Disorder Society Criteria. Mov Disord 32:853–864

Legend: *Within the first three years of disease; CBS: corticobasal syndrome; LD: levodopa; nfaPPA: nonfluent/agrammatic primary progressive aphasia; MSWJ: macro square wave jerks; VOR: vestibulo-ocular reflex; VSGP: vertical supranuclear gaze palsy

### Oculomotor dysfunction domain (O)

*Vertical Supranuclear Gaze Palsy (O1)* has the highest level of diagnostic certainty for the oculomotor dysfunction domain and is identified as a clear limitation of the range of voluntary gaze in the vertical more than in the horizontal plane, affecting both up- and down-gaze, more than expected for age, which is overcome by activation with the vestibulo-ocular reflex; at later stages, the vestibulo-ocular reflex may be lost, or the maneuver prevented by nuchal rigidity.

*Slowing velocity of vertical saccades (O2)* has an intermediate level of diagnostic certainty for oculomotor dysfunction domain and is described as decreased velocity (and amplitude) of vertical greater than horizontal saccadic eye movements established by bedside testing. Gaze should be assessed by command (“Look at the flicking finger”) rather than by pursuit (“Follow my finger”), with the target > 20 degrees from the position of primary gaze. Saccadic movements are slow enough for the examiner to see the eye rotation, rather than just initial and final eye positions in normal subjects. A delay in saccade initiation is not considered slowing. Findings are supported by slowed or absent fast components of vertical optokinetic nystagmus (i.e., only the slow following component may be retained).

*Macro square wave jerks (O3)* have the lowest level of diagnostic certainty for oculomotor dysfunction domain and are defined as rapid involuntary saccadic intrusions during fixation, displacing the eye horizontally from the primary position, and returning it to the target after 200 to 300 milliseconds. Most macro square wave jerks are < 1 degree in amplitude and rare in healthy controls. However, those can be up to 3 or 4 degrees and more frequent (> 10/min) in PSP.

Also *eyelid opening apraxia (O3)* has the lowest level of diagnostic certainty for oculomotor dysfunction domain and is described as the inability to voluntarily initiate eyelid opening after a period of lid closure in the absence of involuntary forced eyelid closure (i.e., blepharospasm).

### Postural instability domain (P)

*Repeated unprovoked falls within the first 3 years of disease (P1)* have the highest level of diagnostic certainty for postural instability domain and are defined as spontaneous loss of balance while standing, or history of more than one unprovoked fall within 3 years after onset of PSP-related features.

*Tendency to fall on the pull test within 3 years of disease (P2)* has an intermediate level of diagnostic certainty for postural instability domain and is defined as susceptibility to fall on the pull test if not caught by examiner within 3 years after onset of PSP-related features. The test examines the response to a quick, forceful pull on the shoulders with the examiner standing behind the patient and the patient standing erect with eyes open and feet comfortably apart and parallel, as described in the item 3.12 of the Movement Disorder Society-sponsored revision of the Unified Parkinson’s disease rating scale (MDS-UPDRS) [7].

*More than two steps backward on the pull test within 3 years of disease (P3)* has the lowest level of diagnostic certainty for postural instability domain and is determined by more than two steps backward, but with unaided recovery, on the pull test, within 3 years after onset of PSP-related features [7].

### Akinesia (A)

*Progressive gait freezing within 3 years of disease (A1)* has the highest level of diagnostic certainty for Akinesia domain and is defined by sudden and transient motor blocks or start hesitation predominant within 3 years after onset of PSP-related symptoms, progressive and not responsive to levodopa.

*Parkinsonism*,* akinetic-rigid*,* predominantly axial and resistant to levodopa (A2)* has an intermediate level of diagnostic certainty for akinesia domain. Levodopa resistance is defined as improvement of the MDS-UPDRS motor scale ≤ 30% with a challenge dose of at least 200 mg or after at least 1000 mg for one month [1].

*Parkinsonism*,* with tremor and/or asymmetric and/or levodopa response (A3)* has the lowest level of diagnostic certainty for akinesia domain.

### Cognitive dysfunction (C)

*A language disorder as nonfluent/agrammatic variant of primary progressive aphasia (C1)* has the highest level of diagnostic certainty for cognitive dysfunction domain and is defined as loss of grammar and/or telegraphic speech or writing progressive aphasia, which has to be persistent (rather than transient).

*A speech disorder as Progressive apraxia of speech (C1)* has the highest level of diagnostic certainty for Cognitive dysfunction domain and is determined by an effortful, halting speech with inconsistent speech sound errors and distortions or slow syllabically segmented prosodic speech patterns with spared single-word comprehension, object knowledge, and word retrieval during sentence repetition, which has to be persistent (rather than transient).

*A frontal cognitive/behavioral presentation (C2)* has an intermediate level of diagnostic certainty for the cognitive function domain and is defined by the presence of at least 3 of the following:

- Apathy: Reduced level of interest, initiative, and spontaneous activity which is clearly apparent to informant or patient;
- Bradyphrenia: Slowed thinking which is clearly apparent to informant or patient;
- Dysexecutive syndrome documented by performance at specific cognitive tests at least 1.5 standard deviations below mean of age- and education-adjusted norms;
- Reduced phonemic verbal fluency documented by a reduction of “D, F, A, or S” words per minute (at least 1.5 standard deviations below mean of age- and education-adjusted norms);
- Impulsivity, disinhibition, perseveration documented by socially inappropriate behaviors, overstuffing the mouth when eating, motor recklessness, applause sign, palilalia, echolalia.

*A corticobasal syndrome (C3)* has the lowest level of diagnostic certainty and is defined by at least one cortical signs among orobuccal or limb apraxia, cortical sensory loss and alien limb and one basal signs among limb rigidity, akinesia or myoclonus. May be simmetric or asymmetric.

### Smartsheet

PSP diagnosis is a dynamic process [8]. Each patient can qualify for multiple phenotypes at different times during the course of the disease. As such the different features of the four core domains can be attributed simultaneously resulting in the fulfillment of different phenotypes at the same visit. The MAX rules were developed to support clinicians in the attribution of a single PSP phenotype as follows: (1) diagnostic certainty; (2) temporal order of onset of symptoms; (3) phenotypic hierarchy with PSP-RS prevailing over any vPSP; (4) MAX hierarchy, with MAX rule 1 prevailing over MAX 2 and 3, and MAX 2 prevailing over MAX 3 [4].

Table 2 summarizes the combination of clinical features allowing the attribution of phenotype and level of diagnostic certainty. MDS diagnostic criteria and MAX rules were used for the smartsheet creation (supplemental material online). By including the presence/absence of each core clinical feature into the smartsheet, each PSP patient can receive the appropriate clinical phenotype and level of diagnostic certainty.

**Table 2:** Combination of the clinical features for the attribution of phenotype and level of diagnostic certainty

Phenotype	Diagnostic certainty	Clinical feature
PSP-RS	Probable	(O1 or O2) + (P1 or P2)
	Possible	O2 + P3
	Suggestive of	O3 + (P2 or P3)
PSP-P	Probable	(O1 or O2) + (A2 or A3)
	Suggestive of	(A2 or A3) + (O3, P1, P2, C1, C2, CC1, CC2, CC3, or CC4)
PSP-PGF	Probable	(O1 or O2) + A1
	Possible	A1
PSP-F	Probable	C2 + (O1 or O2)
	Suggestive of	C2 + (O3 or P3)
PSP-OM	Possible	O1
	Suggestive of	O2 or O3
PSP-SL	Possible	(O1 or O2) + C1
	Suggestive of	C1
PSP-CBS	Possible	(O1 or O2) + C3
	Suggestive of	C3
PSP-PI	Suggestive of	P1 or P2

Modified from Hoglinger GU, Respondek G, Stamelou M et al., for the Movement Disorder Society–endorsed PSP Study Group (2017) Clinical Diagnosis of Progressive Supranuclear Palsy: The Movement Disorder Society Criteria. Mov Disord 32:853–864

Legend: PSP: progressive supranuclear palsy; PSP-CBS: PSP with predominant corticobasal syndrome; PSP-F: PSP with predominant frontal presentation; PSP-OM: PSP with predominant ocular motor dysfunction; PSP-P: PSP with predominant parkinsonism; PSP-PGF: PSP with progressive gait freezing; PSP-PI: PSP with predominant postural instability; PSP-RS: PSP with Richardson’s syndrome; PSP-SL: PSP with predominant speech/language disorder

Video 2 shows how to use the smartsheet for the attribution of clinical phenotype as well as level of diagnostic certainty. Video 3 shows how to use the smartsheet for the application of MAX rule 1 to 4.

The MDS PSP criteria also introduced the category “probable 4R-tauopathy” for joint clinical diagnosis of PSP and corticobasal degeneration [1]. Such criteria showed high specificity and were considered suitable for the recruitment of patients with progressive supranuclear palsy and corticobasal degeneration into therapeutic trials targeting 4R-tauopathy [9]. Video 2 shows how to identify patients affected by “probable 4-repeat (4R)-tauopathy” within the smartsheet.

## Discussion

Herein we provide a practical tool to disseminate the MDS PSP clinical criteria among healthy practitioners and increase confidence in non expert clinicians towards suspicion and diagnosis of PSP.

Our video-guided slide set (Video 1) may serve as a teaching resource for both general neurologists and practitioners, while the smartsheet may represent a valid support in attributing the degree of diagnostic certainty and phenotype based on the identified clinical features (Video 2 and 3).

Reaching a prompt and reliable PSP diagnosis is an urgent need for patients and caregivers as well as for clinicians involved in PSP research. Giving the lack of biomarkers, the clinical diagnosis remains the cornerstone of the diagnostic process. Despite including the complete spectrum of the clinical features displayed by PSP patients, the MDS PSP criteria are complex and not straightforward to be applied for non expert clinicians and in clinical settings [1]. Indeed, there is an urgent need to simplify the MDS PSP criteria and render them accessible to a wider audience of healthy practitioners. Recent evidence on the prodromal phase of the disease shows that PSP patients may disclose soft features way before a diagnosis according to clinical criteria can be attributed [10, 11]. Motor slowing, gait difficulties with postural instability and cognitive and/or behavioral issues either alone or in combination represent among the most common prodromal PSP features reported in the context of primary and secondary care [10, 11]. To anticipate the diagnosis, the MDS PSP criteria introduced a new diagnostic category labelled as “supportive of PSP” [12, 13]. Disseminating the MDS PSP criteria and enhancing awareness of PSP in the context of primary and secondary care would represent an enormous step ahead for a timely identification of the disease in the prodromal stages. Indeed, identifying PSP patients early in the course of the disease would also boost research in the prodromal phase of the disease, which is currently based on large health systems or insurance database [11].

Furthermore, we suggest our tool may be useful also in research settings. Indeed, the slide set may serve as a teaching resource for investigators to render homogeneous the application of the MDS PSP criteria, while the smartsheet may provide a useful guide in attributing degree of diagnostic certainty and phenotype to potential study candidates. To this regard, our smartsheet al.so allows the attribution of the category of “probable 4R-tauopathy”, useful to consider potential candidates for basket-design clinical trials that allow investigation of treatment effects on different clinical syndromes that share the same molecular pathophysiology [14].

We are aware an extensive video-tutorial showing the different clinical features described in the MDS PSP criteria is already available in the literature [15]. However, we believe our proposal represents and independent, added value to the current scenario. First, our videos are embedded in a slide set and accompanied by written and acoustic explanation making our slide set a valid teaching instrument as stand alone. Then, we also provide a smartsheet for the attribution of diagnostic level of certainty and phenotype including the category “probable 4R-tauopathy”, thus simplifying the diagnostic process as much as possible. In keeping with such differences, our tool has been designed to disseminate MDS PSP clinical criteria and increase awareness of PSP diagnosis among non expert clinicians as general neurologists and practitioners.

Finally, our focus was the application of the MDS PSP criteria. We voluntary omitted other signs described as early PSP features but not included in the MDS criteria as the round-the-house sign [16], the zig zag sign [17, 18], the procerous sign or the gunslinger sign [19, 20]. Similarly, we missed to consider the role of imaging supportive findings which lack of a specific role in the attribution of PSP diagnosis and phenotype and, thus, are not considered into the smartsheet [1, 21–24].

## Conclusions

We provide a practical tool to disseminate the MDS PSP clinical criteria among healthy practitioners and increase confidence in non expert clinicians towards suspicion and diagnosis of PSP. Application of our tool may improve early recognition of patients in primary and secondary care and determine a prompt referral to third level movement disorders centers for consideration in clinical trials testing disease-modifying treatments.

## Electronic supplementary material

Below is the link to the electronic supplementary material.

Supplementary Material 1

Supplementary Material 2: Video 1. Video-guided slide set accompanying the operationalized definitions of the core clinical features provided by the MDS PSP criteria summarized in the text and in Table 1.

Supplementary Material 3: Video 2. How to use the the smartsheet for the attribution of diagnosis and clinical phanotype.

Supplementary Material 4: Video 3. How to use the smartsheet for the application of MAX rule 1-4. Supplemental material onle (the smartsheet). Core clinical features (in Grey) for each Functional domain (in Green) can be entered as present (1) or absent (0). In the Blue box Clinical clues can be noted as present (1) or absent (0). Clinical clues do not determine the attribution of the level of diagnostic certainty nor the phenotype. In the Orange box details for the involvement of the Cognitive Dysfunction domain are provided (present?=?1, absent?=?0). Entering the Core clinical features as present/absent determine the automatic attribution of the level of diagnostic accuracy as well as the phenotype as noted in the Pink box. At the bottom of the Pink box the category "Probable 4R-tauopathy" will automatically display YES or NO depending on the phenotype diagnosed. MAX rules are summarized in the Yellow box.

Supplementary Material 5
