# Supplementary material for: A smart tool for non expert clinicians for the dissemination of the MDS criteria for progressive supranuclear palsy
Source: Neurol Sci. 2025 Jan 11;46(5):1959–64. doi: 10.1007/s10072-025-07996-0 (PMC12003616; doi:10.1007/s10072-025-07996-0)
Supplement: Supplementary file 1 — Supplementary Material 1 [file 10072_2025_7996_MOESM1_ESM.docx]

**Video 1.** Video-guided slide set accompanying the operationalized definitions of the core clinical features provided by the MDS PSP criteria summarized in the text and in Table 1.

**Video 2.** How to use the the smartsheet for the attribution of diagnosis and clinical phanotype.

**Video 3.** How to use the smartsheet for the application of MAX rule 1–4.

**Supplemental material onle (the smartsheet)**

Core clinical features (in Grey) for each Functional domain (in Green) can be entered as present (1) or absent (0). In the Blue box Clinical clues can be noted as present (1) or absent (0). Clinical clues do not determine the attribution of the level of diagnostic certainty nor the phenotype. In the Orange box details for the involvement of the Cognitive Dysfunction domain are provided (present = 1, absent = 0). Entering the Core clinical features as present/absent determine the automatic attribution of the level of diagnostic accuracy as well as the phenotype as noted in the Pink box. At the bottom of the Pink box the category “Probable 4R-tauopathy” will automatically display YES or NO depending on the phenotype diagnosed. MAX rules are summarized in the Yellow box.
